# Advances in Wearable Piezoelectric Sensors for Hazardous Workplace Environments

**DOI:** 10.1002/gch2.202300019

**Published:** 2023-04-07

**Authors:** Fatemeh Mokhtari, Zhenxiang Cheng, Chun H Wang, Javad Foroughi

**Affiliations:** ^1^ Carbon Nexus Institute for Frontier Materials Deakin University Geelong Victoria 3216 Australia; ^2^ Faculty of Engineering and Information Sciences University of Wollongong Wollongong NSW 2500 Australia; ^3^ Institute for Superconducting and Electronic Materials University of Wollongong Wollongong NSW 2500 Australia; ^4^ School of Mechanical and Manufacturing Engineering University of New South Wales Sydney NSW 2052 Australia; ^5^ ARC Research Hub for Connected Sensors for Health University of New South Wales Sydney NSW 2052 Australia; ^6^ Department of Thoracic and Cardiovascular Surgery West German Heart and Vascular Center University of Duisburg‐Essen Hufelandstraße 55 45122 Essen Germany

**Keywords:** electronic textiles, energy generators, mining, piezoelectric, self‐powered wearable sensors, vibration, wearable sensors, wearable technologies

## Abstract

Recent advances in wearable energy harvesting technology as solutions to occupational health and safety programs are presented. Workers are often exposed to harmful conditions—especially in the mining and construction industries—where chronic health issues can emerge over time. While wearable sensors technology can aid in early detection and long‐term exposure tracking, powering them and the associated risks are often an impediment for their widespread use, such as the need for frequent charging and battery safety. Repetitive vibration exposure is one such hazard, e.g., whole body vibration, yet it can also provide parasitic energy that can be harvested to power wearable sensors and overcome the battery limitations. This review can critically analyze the vibration effect on workers’ health, the limitations of currently available devices, explore new options for powering different personal protective equipment devices, and discuss opportunities and directions for future research. The recent progress in self‐powered vibration sensors and systems from the perspective of the underlying materials, applications, and fabrication techniques is reviewed. Lastly, the challenges and perspectives are discussed for reference to the researchers who are interested in self‐powered vibration sensors.

## Graphical abstract

Self‐powered piezoelectric sensors are a promising technology to be incorporated into personal protective equipment devices to measure exposure to whole‐body vibration, including hand‐arm vibration syndrome and low back pain. The piezoelectric sensors can help to reduce the risk of these types of injuries and improve the overall health and safety of workers in the mining industry.

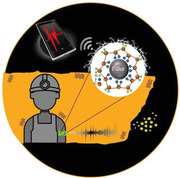

## 1 Introduction

Development and widespread application of internet of things (IoT) technology in sensors over the past decade, provide opportunity for processing and online monitoring to be deployed in our daily life. Sensors are critical devices for remote monitoring of human, structures, and environment.^[^ ^1^ ^]^ The sensors need an accessible and sustainable power source and batteries frequently do not provide the requirement of desirable longevity, thus requiring regular maintenance and therefore ongoing expenses will rise. Powering smart devices with batteries has some challenges including: i) short service life, ii) recycling issue and consequent environmental impact, iii) fires and explosion risk, iv) large size batteries are not desire for small electronic devices. Therefore, wide range of researches are concerned to design and build novel power sources that can convert mechanical into electrical energy to sustain power for smart electronic devices and provide operation durability.^[^ ^2^ ^]^ One of these energy sources is environmental vibration. A popular form of mechanical energy is vibration energy which is available in variety of forms and scales around people's daily life.

Mechanical vibrations, compared with other ambient energy sources available in the environment, contain higher power density that sustains self‐powered sensors.^[^ ^3^ ^]^ Vibration sources are available everywhere from body movement and dynamic motions of industrial machineries, bridges, buildings, vehicles, household appliances, etc.

ISO standard (ISO 2631.1) considers vibration exposures in the frequency range between 0.5 and 80 Hz as harmful to human health. Vibration exposure can cause a range of health problems, including lower back pain, neck pain, headaches, and gastrointestinal track problems. For instance, whole body vibration (WBV) in mining sites has been recognized as one of the major industrial hazards in many mining operations. The vibrations can originate from heavy mining machines such as scrapers, dozers, haul truck, shovels, loaders, load haul dump vehicles, shuttle cars, and most of the earthmoving equipment.^[^ ^4^ ^]^ Operation of mining equipment for a long period of time is often associated with additional ergonomic risk factors such as neck rotation and trunk flexion, lateral bend, and rotation. Long‐term exposure to this type of vibration will reduce comfort and the quality of a coal miner's life through the development of back pain, and adverse consequences for cardiovascular, respiratory, digestive, reproductive, endocrine, and metabolic systems.^[^ ^5^ ^]^

**Figure 1:**
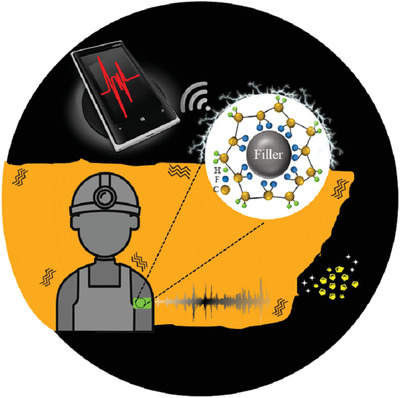
Schematic of a self‐powered piezoelectric sensor for whole‐body vibration monitoring.

Therefore, there is a need to measure this vibration and evaluate exposure levels. Vibration sensors are extensively used in the fields of vehicle and structures safety, health and biomedical equipment,^[^ ^6^ ^]^ intelligent electronic products, and mechanical equipment vibration monitoring. Measurements of whole‐body vibration exposure of workers within underground mines present additional challenges as vibration sensors need batteries as power sources, which can add to fire and explosion risks. One solution to overcome this challenge is developing self‐powered vibration sensors.^[^ ^7^ ^]^ Vibration‐driven environmental energy harvesting (VEH) technologies employ energy‐converting devices to convert environmental vibration energy into electrical energy. Examples of VEH devices include piezoelectric,^[^ ^8^, ^9^ ^]^ electrostatic,^[^ ^10^ ^]^ electromagnetic,^[^ ^11^ ^]^ magnetostrictive,^[^ ^12^ ^]^ and triboelectric.^[^ ^13^ ^]^ Among the VEHs, piezoelectric harvesters appear in more recent research which demonstrates their advantage in structural design and enhancements with respect to their output power and voltage capabilities. VEHs based on piezoelectric materials can be easily integrated to the mechanical oscillators (**Figure** **1**). The vibration energy can be scavenged through the direct piezoelectric effect if the frequency of the environment be close to the resonant frequency of the linear piezoelectric VEHs.^[^ ^14^ ^]^

Piezoelectric energy harvester promotes the fabrication of smart sensors through powering them with energy scavenged from environmental vibrations by means of the piezoelectric direct effect. Piezoelectric materials typically belong to the ferroelectric family and are very popular in sensing and actuation applications due to their functional solid‐state coupling between electrical and mechanical forces. Broadly, piezoelectric materials are categorized based on their structures, compositions, and phases.^[^ ^15^ ^]^ Among the variety of materials exhibiting piezoelectricity, polymers are more considered due to their excellent physical and chemical properties for energy harvesting from ambient vibrations.^[^ ^16^ ^]^ Among the piezoelectric polymers, poly(vinylidene fluoride) (PVDF) is widely used in many wearable applications. PVDF is a semicrystalline polymer which contains five different polymorphic phases (*α*, *β*, *γ*, *δ*, and *ε*). The *β*‐phases of PVDF demonstrate piezoelectric effects.^[^ ^17^ ^]^ The results show that vibration sensing capabilities of PVDF film depend on molecular weight, initial sample thickness, mechanical stretching, and poling.^[^ ^18^ ^]^ It was found that the piezoelectric coefficient (*d*_33_) could be enhanced by decreasing the molecular weight (*M*_w_) of the P(VDF‐TrFE) copolymer, which directly relates to the sensitivity of the sensor.^[^ ^19^ ^]^ The output voltages increased with increasing electrospun PVDF/LiCl web thickness at frequencies below 200 Hz. After increasing the thickness to 350 µm, a reduction in output voltage was reported.^[^ ^20^ ^]^ While many researches have shown that mechanical stretching and poling can enhance the performance of piezoelectric materials, recent research demonstrated that a polar *β*‐phase initiation in PVDF matrix can be achieved by incorporating 5% wt CsPbBr_3_ removing the need for poling and mechanical stretching. Potential application of this composite as vibration sensor was explored under mobile vibration which generated a voltage of 0.8 V.^[^ ^21^ ^]^ By sandwiching the polarized PVDF between two thin copper layers, it was possible to generate a voltage of ≈8 V under a frequency of 30 Hz. Several parameters including vibrational motion, output voltage, and frequency response were modeled based on the equations of motion for a geometrically nonlinear Euler–Bernoulli piezoelectric beam.^[^ ^22^ ^]^

While many research works were done on piezoelectric sensors for energy harvesting from mechanical movement of human body but limited number of research works are done about piezoelectric sensors as personal protective equipment (PPE) device for monitoring safety of mine workers. Due to the importance of vibrational effects on coal miner health and the advantage of piezoelectric materials (especially piezo polymers) in self‐powered sensors, the study toward these kinds of sensors is warranted. Therefore, this review paper explores recent accomplishments in the recent growth in the mining industry and consequently the number of mining workers where their health can be affected by induced vibrations. We discuss the latest research in the area of PPE devices as sensor with a specific aim on polymer‐based piezoelectric nanocomposites. This paper discusses the perspective application and developing sustainable materials for future of power sources from smart textiles.

We also presented a series of piezoelectric sensors in different applications of vibration monitoring and their fabrication techniques. Challenges, opportunities, and guidelines for future development of piezoelectric sensors in hazardous workplace environment are discussed.

## 2 Overview of the Construction and Mining Industry in Australia

**Figure 2:**
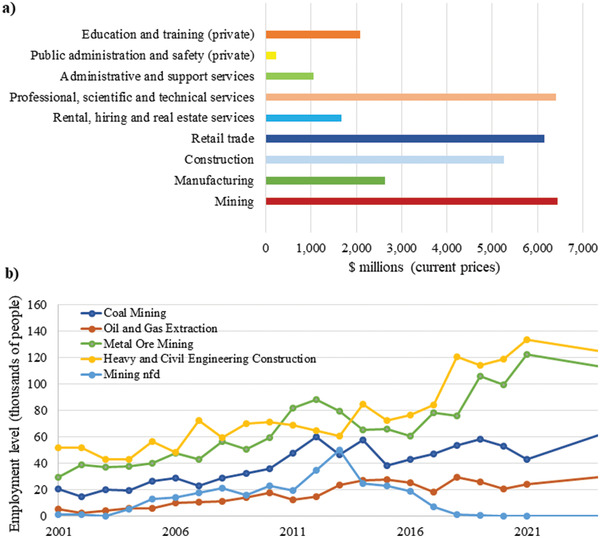
Mining and construction growth in Australia: a) Industry earning movement in 2020–21. The graph was sourced from Australian bureau of statistics. Reproduced with permission.^[^ ^27^ ^]^ Copyright 2023, Australian Bureau of Statistics, b) 2000 to 2021 employment levels and 2025 employment projection for mining and heavy and civil engineering construction industries. The graph was sourced from national industry insight. Reproduced with permission.^[^ ^28^ ^]^ Copyright 2023, Australian Industry and Skills Committee.

Australia is a developed country, and the mining and construction divisions have a significant contribution to Australia's economic growth and development. The construction industry covers a wide range of services including destruction, construction, renovation, and maintenance. The construction industry causes over $360 billion income with the annual growth rate of 2.4% in the next 5 years.^[^ ^23^ ^]^ Australia, the United States, and India are at top list of coal, iron ore, crude steel, bauxite, aluminium, and other significant metals and minerals producer. The mining industry in Australia is explored in this review for the following reasons: i) one of the top countries for mining, ii) it is a country that has a certain amount of regulation regard workers and their health in work environment, and iii) has published public data. The coal mining industry is a major contributor to the Australian economy. The Australian economy affected by sharp growth of the latest mining rise that began in 2003.^[^ ^24^ ^]^ Mining industry in addition to the direct effect on economics is an input in electricity, manufacturing, and petroleum production. **Figure** **2**a shows growth of mining industry in compare with other sectors. The mining industry experienced continued growth of 4.1% ($6.4b) in 2020–21 compared to 2019–20. As can be seen from Figure 2b, employment levels in the mining and heavy and civil engineering construction industry sectors increased in the period of 2007–2014 indicating labor demand created by the resources boom. Based on statistical data, during 2018, mining, drilling, and civil infrastructure combined had employment over 350 000 people.

Due to the fast growth of mining and construction industries and consequently high demand in employment, it is important to consider health and safety of workers in work environment by using the risk management techniques. Unfortunately, the mining workplace is wrapping with different known and unknown hazards that may even cause death. The mining industry has made significant improvements in health and safety over the last decade to decrease fatality rate (per 100 000 workers) from 12.4 to 4.4 workers in 2015 which is lower in compare with other developed countries such as USA and UK. However, the mining industry still has the third highest fatality rate of any industry with an average of nine workers dying each year.^[^ ^25^ ^]^ Based on recent research, mine workers engage in several hazardous activities that are classified in five categories. These categories are the tasks with elevated risk of injury (maintenance, rock and roof bolting, and operating equipment), environmental factors (underground mines, high towers, noisy, poorly ventilated), labors’ characteristics (age and experience), accident types (fall and caught in), and most‐affected body parts ( wrist, hand, and finger).^[^ ^26^ ^]^ The main cause of workers' injuries at mining sites includes body stressing (strains and sprains, back or neck injuries, and tendonitis/tenosynovitis), falling from height, hitting by moving objects, and vehicle incidents. A relatively large percentage of these injuries need long recovery and treatment periods, and some even lead to permanent or partial disability which leads to staff shortage and creates a significant financial problem. One of the solutions for accident preventing in worksites is workers’ safety monitoring by using smart PPE.

### 2.1 PPE and Internet of Thing (IoT)

**Figure 3:**
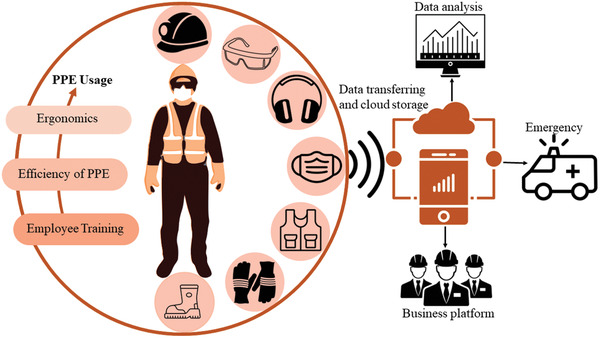
IoT in PPE sensor application.

The mining industry is a mixture of complicated and wide ranges of systems such as driving and transportation system, mining system, air circulation system, electromechanical system, washing and preparation system. Each system includes many processes’ hazards that may lead to accidents and injuries.^[^ ^29^ ^]^ Therefore, there is a need to enhance safety performance and developing mechanism to control and relieve hazards in mining and construction sites. Risk assessment under a proper safety management sector can provide a safe and comfortable working environment for all stakeholders at site. PPE is anything worn by a worker to reduce health and safety risks which includes helmet, safety glasses, ear plugs, heavy working boots, safety gloves, face mask, and body protection (**Figure** **3**).

**Figure 4:**
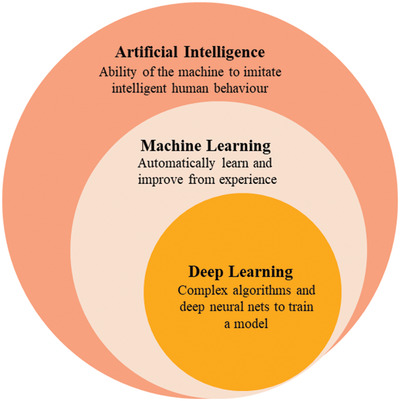
The relation between AI, machine learning, and deep learning in an IoT system. AI creates smart intelligent machines. Machine learning is a subset of AI that helps to build AI‐driven applications and can automatically adapt with minimal human interference. Deep learning is a subset of machine learning that trains a model by using vast volume of data and complex algorithms. Deep learning uses artificial neural networks to mimic the learning process of the human brain.^[^ ^43^ ^]^

It has been stated that factors related to PPE design and efficiency and workers training have effect on PPE usage. Development of training alone may not be sufficient, and it needs to come with design and manufacturing PPE device according to body ergonomic to does not stop user movement in hazardous situation and provide user's comfort.^[^ ^30^ ^]^ The manual health and safety (H&S) monitoring methods in industry is time and energy consuming with a significant delay in report safety issues and use of PPE still remain a concern. One of the ways to optimize operations, improve the quality of performance, and increase the safety of users is collecting data from smart PPE devices. The IoT employ Wi‐Fi technology to monitor, collect, and store data at any stage of production for communication and analysis purposes. IoT provide opportunity for creating new PPE devices with advanced features such as environmental sensing, monitoring, and risk detection and classification. The huge amount of data that are transferred by IoT needs to be collected and organized. In these systems, artificial intelligence (AI) has the role of human to make decision in simple or very complex situations to protect the workers’ lives when they are exposed to high‐risk conditions.^[^ ^31^ ^]^ AI to have higher accuracy in making decision uses machine learning (ML) and deep learning (DL) techniques (**Figure** **4**). DL is considered as a sub‐branch of machine learning, and its applications in dealing with large amounts of data have been successfully demonstrated on many platforms.^[^ ^32^ ^]^ The predictive abilities of ML are being increasingly applied in the field of healthcare. As an example, a wearable medical device‐based system collects details about cardiac patients before and after a heart attack through IoT system, these data based on ML data base, provide platform for a decision support system for disease detection in patients by using DL model.^[^ ^33^ ^]^

The IoT has some challenges and limitation in mining industry including sufficient network coverage, extensive data transfer, and data storage systems.^[^ ^34^ ^]^

Helmet is one of the safety equipment that miners should wear in workplace. Adding Wi‐Fi network and IoT technology to the helmet makes it safer for real‐time environment monitoring in case of air quality, temperature, and humidity.^[^ ^35^ ^]^ Air quality is one of the most important factors to avoid any health issues for mine workers. These harmful gases such as carbon monoxide (CO), carbon dioxide (CO_2_), sulfur dioxide (SO_2_), and nitrous oxides (NO_2_) are usually released during transport, loading, drilling, and waste transferring from the mining site.^[^ ^36^ ^]^ A smart helmet was created to distinguish a risky gas (CO, SO_2_, NO_2_) in the mine. By using the IoT technology, harmful gases were detected, early‐warning sound using buzzer was heard, and data were displayed on an LCD in mining site.^[^ ^37^ ^]^ In another research work, smart helmet was equipped with different sensors to detect the presence of fire, silicosis dust particles, high temperature, harmful gases in both indoor and outdoor environment. In addition to these sensors, GPS tracker was mounted on the helmet to assist in tracking the workers’ current location.^[^ ^38^ ^]^ Vehicle collision is one of the hazard reasons for mining fatalities. According to the report of Western Australian mining industry about vehicle collisions, during 2000–2017, 16 out of 67 number of death were caused by collisions.^[^ ^39^ ^]^ A smart helmet‐based proximity warning systems was designed to simultaneously detect distance between equipment operators and pedestrians in underground mines. Bluetooth beacons are mounted on mining machines including dump trucks, excavators, loaders, and on worker's helmet. As two objects get close to each other smart helmet senses Bluetooth low energy and lights a light‐emitting diode as the alert.^[^ ^40^ ^]^ Noise monitoring and feedback control system is modeled using AI and ML concept. Smart ear muffs or ear plugs are another high‐demand PPE device in mining industry to detect noise above hearing threshold and prevent permanent hearing loss in mine workers.^[^ ^41^ ^]^ An automate PPE‐Tool pair check was developed to notifies the operator and safety officer if the construction or mine worker does not wear the required PPE items. In this system, different sensors (optical, force, touch, photoresistor…) transferred their data by using IoT technology. The results confirm the average time lag of 1.229 s, between actual nonwearing PPE acts and corrective warning reaction which is acceptable to have enough time to alert safety supervisors.^[^ ^42^ ^]^

Overall, PPE and IoT can be used together to enhance workplace safety and productivity. By integrating IoT technology into PPE, workers can receive real‐time feedback on their safety status and environmental conditions and can help organizations to create a safer and healthier work environment by improving situational awareness, reducing accidents, and optimizing productivity.

### 2.2 Smart PPE and Wearable Technologies

Smart PPE is a multidisciplinary approach for the future of occupational health and safety. Smart textiles and wearable electronics devices as PPE can provide sensing and communication technology to enhance safety without impairing the physical performance of workers.^[^ ^44^ ^]^ Recent innovations in e‐textiles and nanotechnology offered possible solutions for the unmet needs of smart PPE design. Nanotechnology covers numerous materials manipulation approaches at scale of less than 100 nm.^[^ ^45^ ^]^ “Wearable technology” as subgroup of e‐textile are terms that describe computers and electronics that are integrated into clothing and other accessories as the body‐worn for measuring, analyzing, and transmitting information.^[^ ^46^ ^]^ Wearable technology contributes a significant part to the IoT, which is the most important element of the Fourth Industrial Revolution (4IR). Based on a report more than $130 billion by 2025 will be assigned to wearable technology and smart textile for creating 4IR in textile and fashion industry.^[^ ^47^ ^]^

Smart PPE in addition to real‐time detection and monitoring of hazardous environment factors, can monitor worker's vital signals and stress level such as heart/breathing rate, body temperature, brainwaves, blood pressure, and muscle bio signals and display information to support, assist, or enhance the capabilities of the worker. The completion of IoT and wearable technology has modernized the field of smart PPE. In order to integrate these PPE sensors to workers’ clothes, they need to have desirable properties like deformability, stretchability, high reliability, and light weight with high‐performance efficiency. Since conventional 3D and 2D electronic devices are rigid and bulky, they cannot meet above requirements. Hence, 1D fiber‐shaped structure can be a good offer for energy harvesting/storage of sensors. The fiber shape sensors are flexible and breathable and can be use in form of twisted, coil, braid, woven, and knitted textile structure.

The common factors for measuring the performance of sensors include output strain/stress, power density, sensitivity, output voltage/current, and cycle life.^[^ ^48^ ^]^ These factors can easily be measured when we have textile structure as sensor.

One of the great candidates for composing smart PPE is polymer‐based piezoelectric materials with fabrication capability of lightweight, low cost, and easy manufacturing. Flexible piezoelectric devices play a key role in wearable energy harvesting and sensor application. Their fabrication has some challenges including high fabrication cost, scalable fabrication, less performance by increasing length and embedding electrodes.^[^ ^49^ ^]^ These challenges require more effort in this direction since smart PPE can minimize accidents in work environment in compare with traditional PPE, and stakeholders are keen to establish smart PPE in their business if it is available in the market at a reasonable cost.^[^ ^50^ ^]^

## 3 Piezoelectric Effect and Piezoelectric Materials

The piezoelectric vibration sensor as a promising technology in sensor filed can be used as self‐powered sensor in PPE devices for mine workers. The PPE device in this specific application needs to be wearable and comfortable for users, therefore prior to select proper fabrication method, it is important to know about piezoelectric effect and different type of piezoelectric materials. Consequently, the manufacturing technics for fabrication of the piezoelectric wearable sensors based on their needs, required sensor sensitivity, and facility accessibility have been reviewed in the following sections.

Piezoelectricity in materials comes from an asymmetric center of the crystalline structure or molecular chain, which can undergo spontaneous polarization or induces polarization under an applied force.^[^ ^51^ ^]^ Direct piezoelectric effect is from dielectric materials that as the result of the mechanical stress, generate electrical charges within their boundaries.

In the converse piezoelectric effect, by applying the electrical field, a mechanical strain occurs. The indirect piezoelectric effect is more relevant to actuators, acoustic devices, and buzzers.^[^ ^52^ ^]^

Direct and converse piezoelectric effects in generating electrical charge or mechanical strain, respectively, are as the result of the ordered arrangement of electric dipoles along the poling direction

(1) $$S_{i}=c_{ij}\;\sigma_{j}+d_{ki}E_{k}\;\left(\mathrm{Converse}\;\mathrm{piezoelectric}\;\mathrm{effect}\right)$$

(2) $$D_{m}=d_{mj}\;\sigma_{j}+\varepsilon_{mk}E_{k}\;\left(\mathrm{Direct}\;\mathrm{piezoelectric}\;\mathrm{effect}\right)$$

The relationship between electric displacement (*D*), electric field (*E*), stain (*S*), and stress (*σ*) are shown in Equations (1) and (2). Here, *c* is the compliance matrix, *d* is the piezoelectric material constant, and *ε* is the dielectric constant matrix. Subscript *m, k*  = 1, 2, 3 and *i, j*  = 1, 2, 3, 4, 5, 6 are assigned to the different direction in Cartesian coordinates.^[^ ^53^ ^]^

The *z*‐axis (direction '3’) indicates polarization direction as the result of poling process.

The rotation motion around axes 1, 2, and 3 and shear motion around axes 4, 5, and 6 which define with subscript “*m*.” It is important to consider that the direction of applying electric field (positive and negative charge) needs to be according to the direction of applying mechanical strain in a piezoelectric energy generator.^[^ ^54^ ^]^

The piezoelectricity of inorganic materials is affected by temperature. Above the Curie temperature, a material dissipates its piezoelectric and ferroelectric properties. Piezoelectricity in organic materials comes from molecular dipoles reorientation under an applied stress that results in dipoles alignment in a particular direction that produces by a net polarization.^[^ ^55^ ^]^ In general, piezoelectric materials can be categorized into three sections: piezoelectric ceramics, single crystals (SCs), and polymers.

### 3.1 Piezoelectric Ceramics

Piezoceramics owing to their outstanding dielectric, piezoelectric, and electrical properties, have the key role in the advancement of modern technologies.^[^ ^56^ ^]^

Nanowires, bulk, and nanofibers are different forms of piezoceramic. The excellent characteristics are highlighted property of the bulk form even they do not have flexibility. The common piezo‐ceramic is lead zirconate titanate (PZT) which contains 60% lead and causes damage to the human kidneys, brain, and the nervous system; it is also harmful to the surrounding environment.^[^ ^57^ ^]^

The European Union in 2003 classified PZT as a hazardous material that need to be replaced with safer options. These demands increased research interest to find lead‐free piezoceramics such as potassium sodium niobate (K, Na)NbO_3_ (KNN)‐based, sodium bismuth titanate (Bi_0.5_, Na0.5)TiO_3_ (BNT)‐based, BaTiO_3_ (BT)‐based, and BiFeO_3_ (BFO)‐based piezoelectrics.^[^ ^58^ ^]^

### 3.2 Single Crystals

The general formula for lead‐based relaxor materials is Pb(B_1_, B_2_)O_3,_ where B_1_ presents low‐valence cation (Zn^2+^, Mg^2+^, Ni^2+^, Yb^3+^, In^3+^, or Sc^3+^) and B_2_ assigned to high‐valence cation (Nb^5+^, Ta^5+^, or W^6+^). The solid solutions between a relaxor and lead titanate PbTiO_3_ (PT) have a morphotropic phase boundary region separating the rhombohedral (R) and tetragonal (T) phases and there are also boundaries between orthorhombic (O) and monoclinic (M) phases.^[^ ^59^ ^]^ In recent 20 years, the SCs of the relaxor PT owing to their high transition temperatures, low density, large piezoelectric coefficients, and the nontoxic chemical composition have been widely explored.^[^ ^60^ ^]^ The lead magnesium niobate and lead titanate (PMN‐PT) as the piezoelectric SC can have the piezoelectric constant (*d*_33_) up to 2000 pC N^−1^ which is four to six times higher than PZT piezoelectric ceramic.^[^ ^61^ ^]^ Domain engineering by manipulating the domain structure through patterned electrodes^[^ ^62^ ^]^ and poling methods^[^ ^63^ ^]^ enhances the piezoelectric performance of relaxor‐PT SCs. This technique leads to have superior piezoelectric properties in SCs in compare with ferroelectric ceramics.

### 3.3 Piezoelectric Polymers

Compared to traditional piezoceramic materials, polymer piezoelectric has unique advantages such as being lightweight, ease of processing, and super‐flexibility, that makes them ideal to be used in flexible and comfortable wearable device.^[^ ^64^ ^]^ However, piezoelectric polymers demonstrate low piezoelectric coefficients in comparison with piezoceramics.^[^ ^65^ ^]^

Polymers such as polyureas, fluoropolymers polypeptides, polyamides, polysaccharides, and polyesters have piezoelectric properties. A wide variety of biopolymer materials including cellulose, collagen, and silk also exhibit piezoelectric behavior.^[^ ^66^ ^]^ PVDF is the most common piezoelectric polymers for sensor and actuator applications. PVDF has various desirable properties, including mechanical strength, biocompatibility, thermal, and chemical stability. The crystallinity of PVDF has significant role in its piezoelectric performance, optical, mechanical, electrical, and thermal properties.^[^ ^57^ ^]^ PVDF has four crystalline forms including *α*, *β*, *γ*, and *δ*‐phase. Among these, the nonpolar *α*‐phase is thermodynamically promising, however it needs to be converted to polar *β*‐form for a prevailing ferroelectricity behavior. To achieve better ferro‐ and piezoelectric response from PVDF, the higher crystallinity degree (*β*‐phase formation) is needed. Numerous techniques including mechanical stretching, annealing, adding filler, and poling are required to enhance PVDF crystallinity.^[^ ^67^ ^]^ The primary piezoelectric properties of polymers are listed in **Table** **1**.

**Table 1:** Piezoelectric properties of polymers^[^ ^68^ ^]^

Polymer	Piezoelectric coefficients *d_ij_* [pC N^−1^]			Electromechanical coupling coefficients		Relative permittivity [*ε*_r_]
	*d*_33_	*d*_31_	*d*_14_	*k*_33_	*k*_31_	
PVDF	25–35	8–22	–	0.2	0.12	6–12
P(VDF‐TrFE)	24–40	12–25	–	0.29	0.16	18
P(VDF‐CTFE)	140	–	–	0.36	–	13
P(VDF‐HFP)	24	30–43	–	0.36	0.187	11
Polyamide 11	4	14–100	–	–	0.049	5
Polyimide	2.5–16.5	–	–	0.048–0.15	–	4
Polylactic acid (PLA)	–	1.58	9.82	–	–	3–4
Cellulose	5.7	1.88–30.6	−35 to 60	–	–	–
Polyurethane	–	27.2	–	–	–	4.8–6.8
Polyurea	19–21	10	–	–	0.08	–
Polyacrylonitrile (PAN)	–	2	–	–	–	–
Parylene‐C	2	–	–	0.02	–	–
Liquid crystal polymers	−70	–	–	–	–	–

## 4 Piezoelectric Sensors in Vibration Monitoring

**Figure 5:**
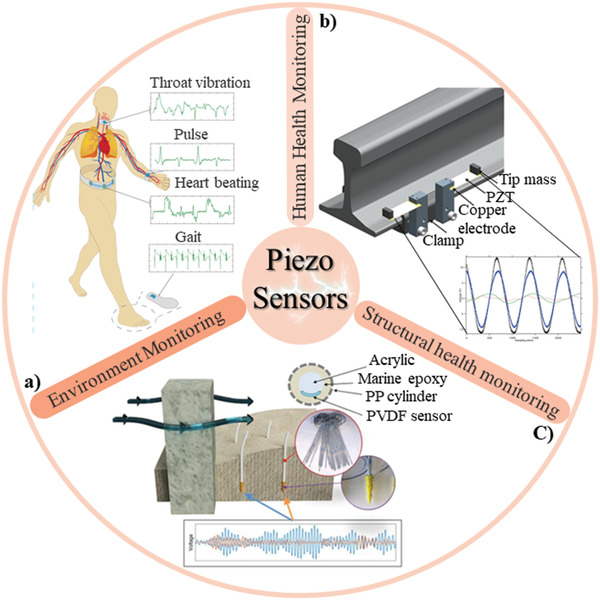
Application of piezoelectric vibration sensors in three different fields: a) The schematic diagram of piezoelectric sensors placed on the throat, wrist, abdomen, and sole of the feet to monitor physiological microvibration signals. Reproduced with permission.^[^ ^72^ ^]^ Copyright 2022, Elsevier, b) a clamped cantilevered piezoelectric on a railway track. Reproduced with permission.^[^ ^73^ ^]^ Copyright 2016, Extrica, c) during scour, piezo‐rod sensors detects frequency variation. Reproduced with permission.^[^ ^74^ ^]^ Copyright 2021, SAGE.

Piezoelectric materials showed their capability in vibration monitoring in a wide range of applications including environmental and structural health monitoring. Based on their excellent performance, piezoelectric materials can be considered as reliable wearable vibration sensor in mining workplace as well. While analyzing vibration in piezoelectrics, it is important to have proper vibration measuring system. Vibration measurement includes measuring various parameters such as acceleration, displacement, and velocity. Accelerometer is the most common device to measure vibration with diversity in design, size, and range. An accelerometer responds to changes in its inertia and can put the dynamic acceleration proportionally as a voltage output. Accelerometers based on principle function are categorized in various types (piezoelectric, piezoresistance, and capacitive).^[^ ^69^ ^]^ An acceleration sensor is a critical element in vibration monitoring, which can be used in a variety of applications, e.g., human health monitoring (finger touching, muscle behavior, vocal cord vibration, and so on), environment monitoring (earthquake monitoring, gas sensor, liquid flow, wind flow), and structural health monitoring (vehicle safety, railway monitoring, bridge monitoring) (**Figure** **5**). Among different types of accelerometers, piezoelectric sensors can be self‐powered, while the electric output is very small and could be influenced by the environmental noise that creates huge opportunities across various end‐user sectors.^[^ ^70^ ^]^ Application of wearable piezoelectric vibration sensor as PPE device for mine work environment is considerable in two aspects: 1) detecting vibration signal and monitoring them to evaluate workers; health conditions, 2) using vibration stimulation as a power source to charge low‐power electronic devices of worker without the need of batteries. The global market for piezoelectric sensors is forecast to reach $1.9 billion by 2026, with a compound annual growth rate of 4.5% from 2021 to 2026.^[^ ^71^ ^]^

### 4.1 Human Health Monitoring Systems (HHMS)

HHMS attract recent research interest for their broad prospects in preventive healthcare. The important concern about HHMS’ sensors is their high flexibility and stretchability to monitor vital health factors and identify physical movements. The rapid growth of wearable and implantable electronic devices leads to receive great interest for sensors with high durability and accuracy. Recently, mechanical vibrations are consider by researchers as the power source for sensors and wireless electronics in a wide variety of applications including microelectro mechanical systemsMEMS, health monitoring, and wearable sensors.^[^ ^75^ ^]^ Wearable technologies and biomedical devices have wide range applications in medical diagnostics, precision therapy, and real‐time health tracking, such as respiration, heart beats, and joint motion.^[^ ^76^ ^]^ The long‐term vision for wearable technology is to develop them to a point that can be integrated into everyday clothes, which allow nonprofessional users to constantly monitor their physiological signals everywhere without the need of any expensive devices. Vibrations or regular mechanical pulses occur frequently in the body which include the chest and abdomen vibration that caused by breathing and heart beating, blood shock vibration, and the throat vibration during talking or snoring. These vibrations carry important physiological information and can be known as physiological microvibration signals (PMVS). Monitoring of PMVS can have significant effects in terms of early diagnosis for treating disease such as sleep apnea disorder. PMVS were successfully fabricated by PVDF‐TrFE/nanoclay electrospinning nanofibers for monitoring different part of human body vibration (Figure 5a).^[^ ^72^ ^]^ Piezoelectric materials, especially piezo polymers such as PVDF, offer good possibility to be used in PPE as wearable vibration sensor. These materials can detect environmental vibration and also any pressure or deformation that comes from human biomechanical activities^[^ ^77^, ^78^, ^79^ ^]^ including breathing,^[^ ^80^, ^81^ ^]^ blinking, finger movement, and gait,^[^ ^82^ ^]^ which made them suitable candidate for health monitoring of mine workers through smart PPE.

PPE as the sensor plays a significant role in fulfilling the requirement of safety in a range of environmental sensing, monitoring, and risk identification for mine workers. **Table** **2** shows some examples of PPE sensor used as applications.

**Table 2:** Piezoelectric PPE sensors for safety of industry workers

Protection part	Sensor functionality	Materials	Sensor image	Ref.
Head	By using sensor and IoT, it is possible monitor wearing helmet at work site by registered person	thermal IR sensor	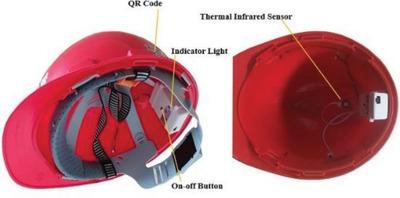	(Reproduced with permission.^[^ ^83^ ^]^ Copyright 2022, Wiley)
Head	Measuring the pressure on the head when wearing protective equipment	Flexible piezoelectric rubber	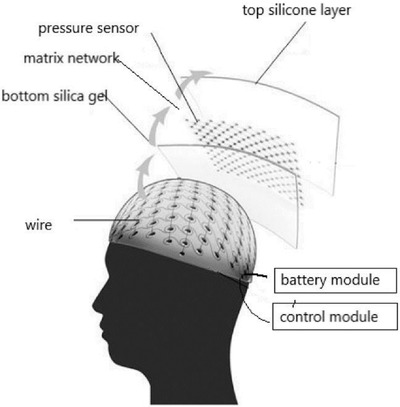	(Adapted under the terms of the CC‐BY Creative Commons Attribution license.^[^ ^84^ ^]^ Copyright 2019, The authors published by MDPI.
Head	Energy harvester and wireless human motion sensor	Al‐doped ZnSnO_3_–PDMS film, sandwiched between two layers of thin copper tape	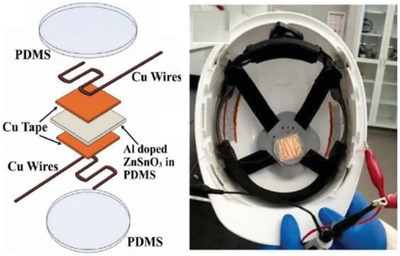	(Reproduced with permission.^[^ ^85^ ^]^ Copyright 2022, Elsevier)
Head	Harvesting vibration energy from human motion to power a wireless pedometer for real‐time transmitting data reporting to a mobile phone.	Wavy‐structure Al‐Kapton‐Al films are sandwiched between Al back‐coated polytetrafluoroethylene (PTFE) thin films as the sensor	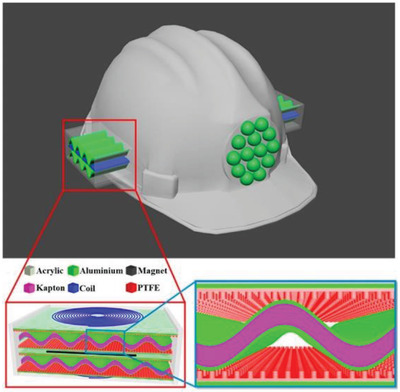	(Reproduced with permission.^[^ ^86^ ^]^ Copyright 2016, ACS)
Eyes	Monitoring eyelid motion and eye fatigue	PZT ribbon	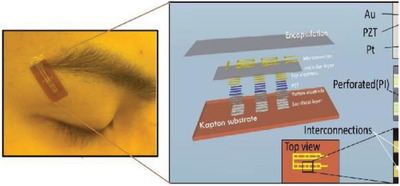	(Reproduced with permission.^[^ ^87^ ^]^ Copyright 2018, IOP)
Eyes	Due to light stimulation, electrical charge will be generated in retina by photoreceptors	Graphene‐based full‐field electroretinogram (ERG) sensor embedded in a contact lens	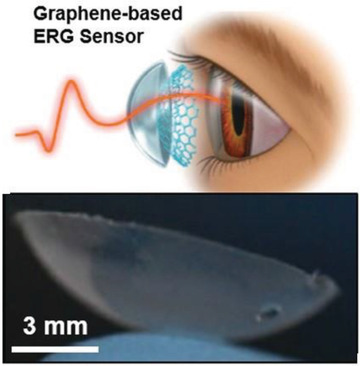	(Reproduced with permission.^[^ ^88^ ^]^ Copyright 2019, Wiley)
Eyes	Monitoring of eye‐blink and movement	Honeycomb printed graphene	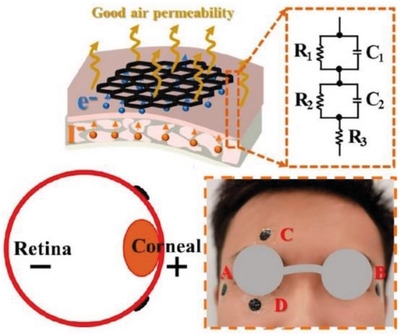	(Reproduced with permission.^[^ ^89^ ^]^ Copyright 2022, ACS)
Eyes	Self‐powered eye motion sensor	PDMS+PEDOT:PSS	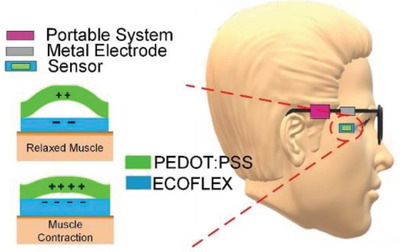	(Reproduced with permission.^[^ ^90^ ^]^ Copyright 2020, Elsevier)
Ears	Energy harvesting from jaw movements	PZT	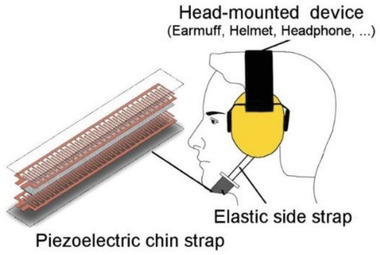	(Reproduced with permission.^[^ ^91^ ^]^ Copyright 2014, IOP)
Ears	Energy harvesting from jaw movements	PVDF	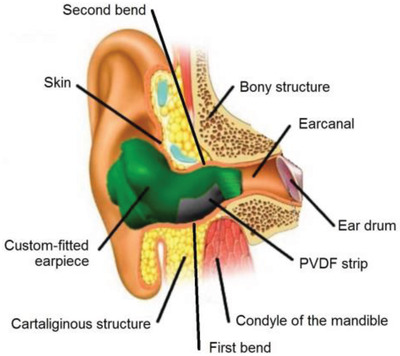	(Reproduced with permission.^[^ ^92^ ^]^ Copyright 2018, IEEE)
Foot	Energy harvesting by walking through sensor placed in shoe	Piezoelectric PZT ceramic	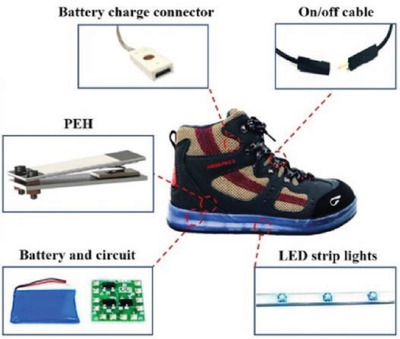	(Reproduced with permission.^[^ ^93^ ^]^ Copyright 2019, Elsevier)
Foot	Harvesting and storage of walking energy	TiO_2_ nanotube (NT)+PVDF film	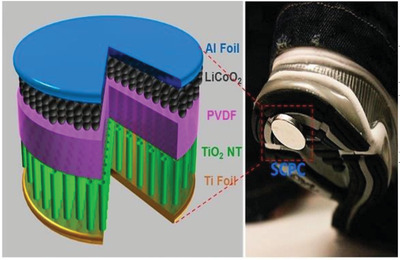	(Reproduced with permission.^[^ ^94^ ^]^ Copyright 2012, ACS)
Foot	Harvesting energy from walking or running	Piezoelectric sensor (PZT)	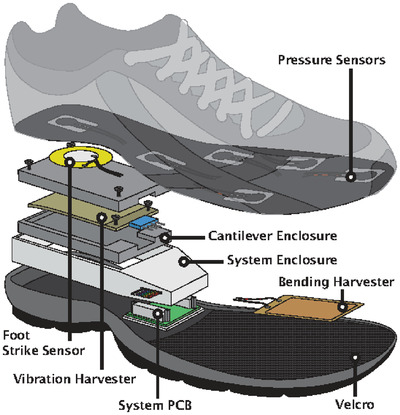	(Reproduced with permission.^[^ ^95^ ^]^ Copyright 2014, IEEE)
Foot	Harvesting of random and irregular vibrational energy from the human foot	Hybrid triboelectric‐piezoelectric (PVDF) nanogenerator (TP‐NG)	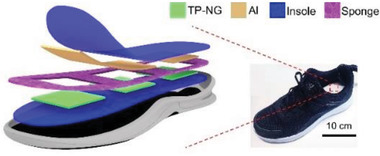	(Reproduced with permission.^[^ ^96^ ^]^ Copyright 2020, Elsevier)
Hands	A self‐powered wearable glove that harvests and manages the biomechanical energy of the fingers	PVDF film	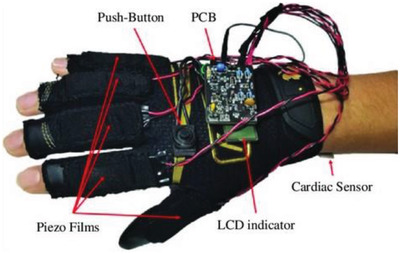	(Reproduced with permission.^[^ ^97^ ^]^ Copyright 2021, Wiley)
Hands	Hand movement diagnostics utilizing IoT technologies	Conductive fiber coated with PVDF and then Ag NWs spray coated on top	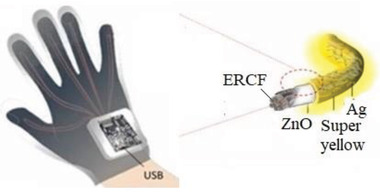	(Reproduced with permission.^[^ ^98^ ^]^ Copyright 2022, Elsevier)
Hands	Generate pressure maps in real‐time while human hand grabbing different objects in daily life	Polyimide surfaced that AG nanoparticles printed in pattern on it	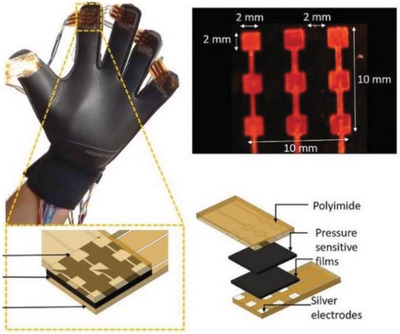	(Reproduced with permission.^[^ ^99^ ^]^ Copyright 2022, Wiley)
Body	All‐fiber piezoelectric/triboelectric generator for wearable gesture monitoring	electrospinning silk fibroin and PVDF nanofibers on conductive fabrics	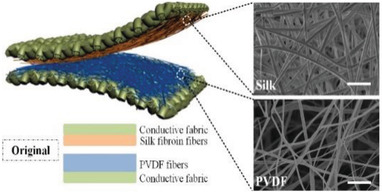	(Reproduced with permission.^[^ ^100^ ^]^ Copyright 2018, Elsevier)
Body	All‐fiber nanogenerator for wearable sensor	Pt‐PVDF nanofiber	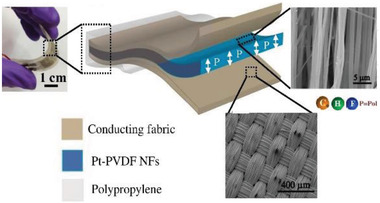	(Reproduced with permission.^[^ ^101^ ^]^ Copyright 2018, Elsevier)
Body	All fiber human movement sensor	NaNbO_3_/PVDF composite nanofibers	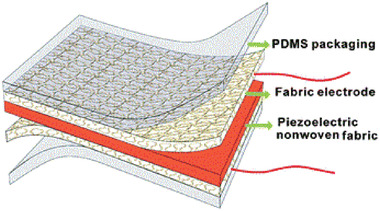	(Reproduced with permission.^[^ ^102^ ^]^ Copyright 2013, Royal Society of Chemistry)
Body	Multilocal strain (MLS) sensor for harvesting energy from human in vibration mode (voice, respiration, blood pulse) and compression mode (body movement, pressure of fingertip and gait, neck and joint movement (elbow and knee..)	Knitted polyester multifilament +PVDF film	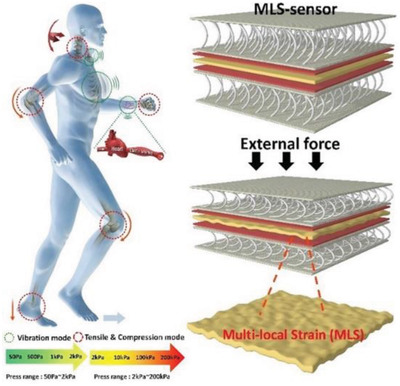	(Reproduced with permission.^[^ ^103^ ^]^ Copyright 2020, Elsevier)
Body	Lightweight wearable cooling and dehumidifying system for 6 h operation without taking off the PPE	knitted polyester fabric + 3D printing with aluminum alloy+ resin+fan	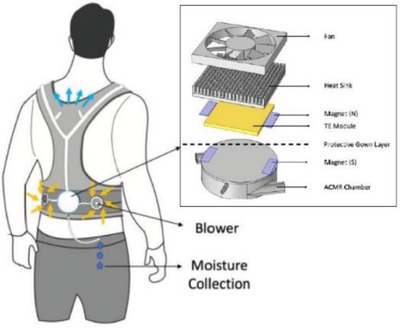	(Reproduced with permission.^[^ ^104^ ^]^ Copyright 2022, Elsevier)
Respirator	Providing optimal reliability and resistance against the wet and warm atmosphere around the face mask during harvesting energy from face masks	encapsulation based on PVDF, PDMS, CA, and parylene C	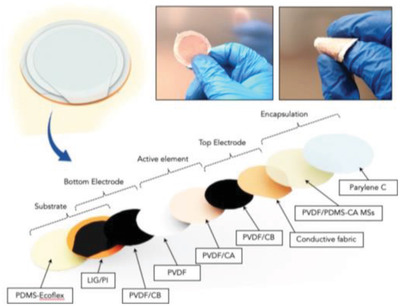	(Reproduced with permission.^[^ ^105^ ^]^ Copyright 2021, ACS)
Respirator	Mechanical and sound waves sensor for daily speaking with capability of wearing time of face masks	Melt blowing of P(VDF‐TrFE)	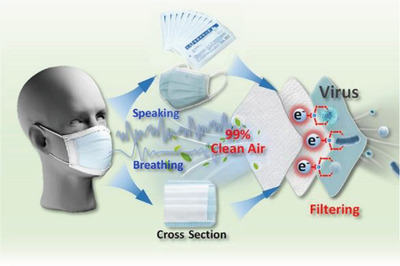	(Reproduced with permission.^[^ ^106^ ^]^ Copyright 2022, ACS)
Respirator	Smart face mask for wireless breath monitoring	Au/parylene/Teflon AF films	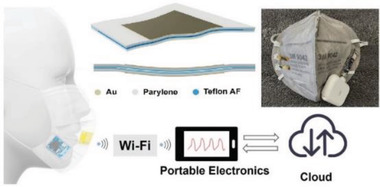	(Reproduced with permission.^[^ ^107^ ^]^ Copyright 2022, Wiley)
Respirator	Photodynamic/photothermal antimicrobial face mask	electroactive polymer PVDF‐HFP as the matrix+TTVB as the dopant	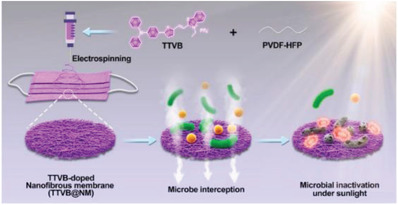	(Reproduced with permission.^[^ ^108^ ^]^ Copyright 2021, Elsevier)

### 4.2 Structural Health Monitoring (SHM)

SHM is a technique to detect damage based on vibrational response from the structure and identify deviation from optimal working condition. Mass, stiffness, and damping value are affected and changed in a damaged structure.^[^ ^109^ ^]^ This change can be detected by variations in structure's natural frequency, mode shape, and modal strain energy. SHM technique is widely used in the field of mechanical engineering structures (rotating machinery, pipeline, wind turbine, railway), aerospace and civil (bridges, buildings). Continually monitoring of the structures increases their lifetime and public safety and consequently reduces maintenance and renewal cost. Robust SHM methods are developed for continuous monitoring, acquisition, validation, and analysis of technical data to simplify life‐cycle management decisions.^[^ ^110^, ^111^ ^]^

Vibration energy harvester based on piezoelectric material exhibited their capability for converting ambient vibration energy to electrical energy in railway (even for underground subway) health monitoring.^[^ ^112^ ^]^ Hence, railway is one of the most important transportation services for passengers and cargoes, it has extensive effect on human society. Railway vibration is usually wasted as heat dissipated by shock absorbers or rubber pads. In the railway systems, for reducing the maintenance cost and inspection obligations, self‐power wireless sensors are highly desirable for health monitoring. A rail‐borne seismic piezoelectric energy transducer was fabricated to utilize railway acceleration differences in a small amplitude of 0.2 to 0.4 mm and generating electricity at a low‐frequency range of 5 to 7 Hz (Figure 5b).^[^ ^113^ ^]^ Currently piezoelectric sensor is one of the most demanding vibration sensors for structural monitoring in the industry. This wide use of application in different frequency ranges confirms their capability to be used in wearable vibration sensor for mine workers' health monitoring as well.

### 4.3 Environment Monitoring

As another example of current use of piezoelectric materials in vibration monitoring is their application in environmental monitoring. In the harsh environmental condition (underground or subsea), environmental monitoring sensors are needed to detect any change of industrial facilities including pressure and temperature. This self‐powering sensory device demonstrated its great potential for monitoring the surrounding environment, disaster warning, and meteorological record in IoT applications. Piezoelectric sensors have a broad application prospect in the field of environmental monitoring (**Table** **3**). In recent years, these sensors can store small‐scale energy effectively for monitoring many important environmental parameters such as temperature, humidity, rain, wind speed, water quality, wave, and light intensity. During a flood, reduction of bridge structure capacity due to the scour failure leads to bridges' collapse without warning. Bridges' collapse is a worldwide public concern, therefore it is essential to real‐time monitoring of scour‐depth variations and prevent catastrophic collapse that brings significant operational problems and death.^[^ ^125^ ^]^ Piezoelectric‐driven rod scour sensors excited by vortex shedding were presented to collect data from scour depth (Figure 5c). The piezo‐rod was designed for operation in harsh environment especially when flow of air and muddy water alter other measurement systems including magnetic sliding collars, float‐out devices, tilt sensors, and robust time‐domain reflectometry.^[^ ^74^ ^]^

**Table 3:** Piezoelectric vibration sensor's performance in SHM applications

Material	Application	Frequency	Power/voltage output	Dimension	References
PZT sheet	Harvesting vibration of rolling bearings	15 Hz	60–131 µW, 25 V	47 × 50.2 × 10 mm	ref. [114]
PVDF sheet	Scavenge 2D vibration energy and monitor the vibration direction.	61.5 Hz	6.5–7.5 V	30 × 12 × 0.085 mm	ref. [115]
PZT	Structural health monitoring	0–200 Hz	–	10 × 10 × 0.3 mm	ref. [116]
PVDF	Detection of pipe leakage	2 kHz	≈2 V	–	ref. [117]
PVDF/carbon dots nanofibers mat	Sensitive detection of low‐frequency small accelerations	1.6 Hz	2.1 µA cm^−3^, 55.6 V cm^−3^	–	ref. [118]
PZT sheet	Harvesting bridge vibration	60 Hz	1.59 mW	0.3 × 50 × 27.8 mm	ref. [119]
Polytetrafluoroethylene (PTFE)	Rail track fracture detection	3–133 kHz	1 to 10 V	150 cm × 150 cm × 0.5 cm	ref. [120]
PVDF‐TrFE coating	0BStructural health monitoring	100 kHz	10 V	–	ref. [121]
PVDF yarn	1Structural damage monitoring	4 Hz	1 V	–	ref. [122]
PAN/BaTiO_3_	4B Structural health monitoring	1 Hz	3.2 V	80 mm × 15 mm	ref. [123]
3BPVDF/ZnO porous FILM	4BStructural health monitoring	30 Hz	0.46 mW	3 cm in diameter	ref. [124]

**Figure 6:**
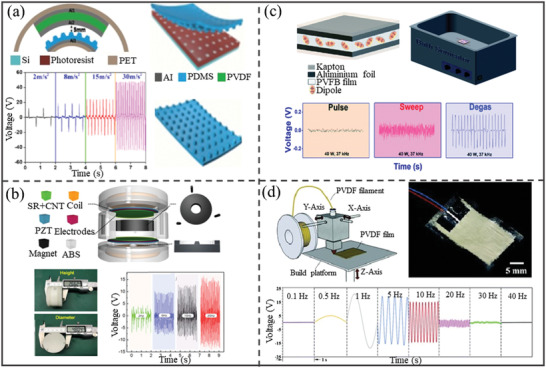
Structural schematic of different types of piezoelectric vibration sensors: a) fabrication sketch diagram of the PDMS film with patterned microstructure and its open‐circuit voltages of the enhanced piezoelectric unit with a vibration amplitude of 6 mm. Reproduced with permission.^[^ ^126^ ^]^ Copyright 2018, Wiley. b) Triboelectric‐piezoelectric‐electromagnetic hybrid nanogenerator structure and its open‐circuit voltage at different frequencies. Reproduced with permission.^[^ ^127^ ^]^ Copyright 2018, Elsevier. c) Schematic diagram of the 30% PVFB PENG device attached to the bath sonicator under varying power and sonicator operation modes. Reproduced with permission.^[^ ^128^ ^]^ Copyright 2022, Royal Society of Chemistry. d) Fabrication of the piezoelectric PVDF films using FFF and voltage generated by a polarized printed piezoelectric PVDF sensor at different actuating frequencies of 0.1, 0.5, 1, 5, 10, 20, 30, and 40 Hz. Reproduced with permission.^[^ ^129^ ^]^ Copyright 2022, Royal Society of Chemistry.

Due to a wide application range of piezoelectric sensors, much research works focus on achieving high sensitivity and power output. The nanogenerator film was made from PVDF film, electrodes, and a layer of polydimethylsiloxane (PDMS) film as the self‐powered vibration sensor. The double‐arched structure of the sensor led to sense a vibration amplitude of 6 mm with sensitivity of 2.21 V g^−1^ and linearity error of 5.91%. Furthermore, the sensor demonstrated not only stable repeatability and long‐term stability but also consistent output performance even after being soaked in water at temperatures as high as 70 °C (**Figure** **6**a).^[^ ^126^ ^]^ A vibration energy harvesting device was fabricated from acrylonitrile butadiene styrene copolymers and 3D‐printing technique for wireless monitoring application with a peak output power of 122 mW. This device with weight of 80 g and small dimension of Φ48 mm × 27 mm is useful for IoT and the intelligent mobile terminal. The high sensitivity and lower energy loss of this sensor lead to use it in application such as the slapping desk vibration and the running car vibration for small energy harvesting (Figure 6b).^[^ ^127^ ^]^ An acceleration sensor made from liquid metal mercury droplet and PVDF nanofiber film shows a wide detection range from 0 to 60 m s^−2^ with a high sensitivity of 0.26 V s m^−2^ and excellent durability over 200 000 cycles.^[^ ^70^ ^]^ Highly crystalline PVDF films with the loading of 30% BNT NC filler under controlled humidity (60% RH) and solvent evaporation conditions were fabricated as the vibration sensor with an instant power density of 11.8 mW m^−2^. The bath sonicator which provided the ultrasonic vibration under three modes of operation (pulse, sweep, and degas modes) was used to induce vibration to the sample. The device was utilized to convert sonicator‐induced vibration, resulting in an output of 1.2 V/20 nA for the sonicator in degas mode (Figure 6c).^[^ ^128^ ^]^ The fused filament fabrication (FFF) technique and two post‐treatments process including mechanical stretching and electrical poling were used to fabricate PVDF film for vibration monitoring. The *d*_33_ value of the fabricated film has been substantially enhanced by ≈10–100 times higher than the printed film without doing any post‐treatment. As the frequency increased from 0.5 to 10 Hz, the peak‐to peak voltage increases from 9 to 39 mV and then by increasing frequency up to 30 Hz the voltage decreases to 2 mV. The fabrication of piezoelectric sensors will become simpler with the use of this multimaterial 3D‐printing technique, eliminating the necessity for post‐metallization processes (Figure 6d).^[^ ^129^ ^]^

## 5 Fabrication Techniques of Piezoelectric Sensors

The key challenge for mass production of wearable piezoelectric sensor is finding approach to fabricate them by existing textile machine. This approach will provide low cost and most convenient way to integrate these sensors to everyday clothes. The desire properties for wearable electronic devices are providing comfort, long lifetime, ease of fabrication process and integration to everyday clothes, and supplying proper power output. Piezoelectric film fabrication techniques including tape‐casting, screen‐printing, composite sol–gel, aerosol deposition, electrophoretic deposition, and ink‐jet printing make films with thicknesses of <50 µm with the same properties of bulk piezoelectric materials. Piezoelectric fibers are fabricated through wet, dry, melt, and electrospinning. 3D structures of piezoelectric sensors are mostly fabricated by FFF and stereolithography (SLA) techniques. Here, we describe three common methods for fabricating the active sensitive layer of piezoelectric sensors.

### 5.1 3D Printing

Additive manufacturing, colloquially known as 3D printing, enables the manufacturing of customized parts with complex geometries using a variety of materials such as polymers, ceramics, and metals without the need for molds or formative and subtractive manufacturing processes.^[^ ^130^ ^]^ In recent years, 3D‐printing technology based on computer aided design modeling has been developed to fabricate wearable piezoelectric generators due to its structural flexibility, design accuracy, and rapid prototyping capability.^[^ ^131^ ^]^ Polymers, ceramics, and composite are three category of materials that are used for 3D printing of piezoelectric materials.^[^ ^132^ ^]^ The most piezoelectric polymers that are used for 3D printing include polymers such as PVDF, polycarbonate, nylons, polyacrylonitrile, poly(vinylidene cyanide‐vinyl acetate), poly(phenyl ether nitrile), PZT, PMN‐PT, and polyvinyl chloride. The functionalities of piezo generators are highly dependent on the structure of the device, such as the rotary mode for printed rotating sleeves.^[^ ^133^ ^]^ The mechanical response of a piezoelectric generator is dependent on the 3D structure. To enhance the piezoelectric performance in the direction of stress, the effect of structures “nanoconfinement” including holes and arrays can be carried out to improve directional arrangement of dipole moments, uniform distribution of compressive stress, and suppress strain in the direction of vertical stress.^[^ ^134^ ^]^ It was proven that the honeycomb structure can affect the response frequency of piezoelectric generator. In the honeycomb structure, by changing mass and stiffness of substrate, the flexibility of the piezoelectric structure will be affected which significantly affects its power output.^[^ ^135^ ^]^

The extrusion‐based printing technology has the capability to fabricate high‐precision piezoelectric porous parts with superior electromechanical performance. However, the method for achieving high content of polar phase in piezoelectric part is a big challenge for 3D‐printing technique.^[^ ^136^ ^]^ Since piezoelectric materials need poling processes to enhance their properties (corona poling or contact poling), poling can be done during 3D printing which is known as in situ poling or can be done as the posttreatment process.^[^ ^137^ ^]^ 3D‐printing technology as an efficient and intelligent manufacturing technology is a crucial method for the development of smart technologies based on piezoelectric materials.

**Figure 7:**
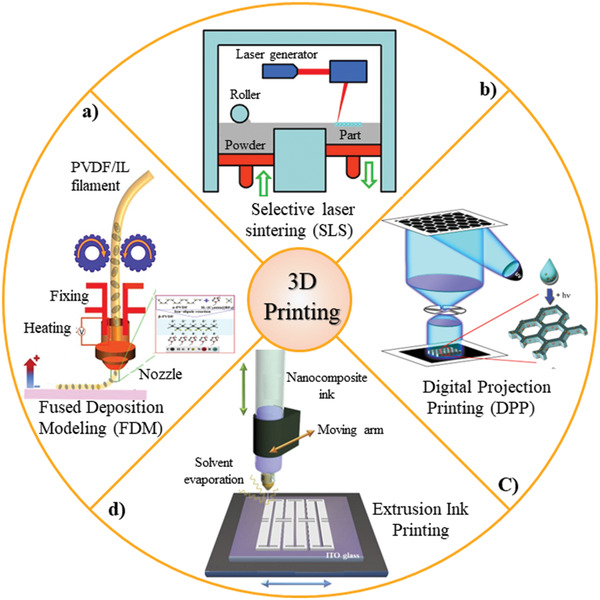
a) FDM printing of the PVDF piezoelectric device. Reproduced with permission.^[^ ^138^ ^]^ Copyright 2021, ACS. b) SLS 3D printing of polyvinylidene fluoride (PVDF)/graphene nanocomposite. Reproduced with permission.^[^ ^139^ ^]^ Copyright 2021, Elsevier. c) Schematic of the function of DPP printer: digitized pattern projected onto the piezoelectric composite solution and solidify by exposing to the light. Reproduced with permission.^[^ ^141^ ^]^ Copyright 2014, ACS. d) 3D printing of piezoelectric BaTiO_3_ NP/P(VDF‐TrFE) ink on a piece of ITO glass that acts as the bottom electrode during the poling process. Reproduced with permission.^[^ ^142^ ^]^ Copyright 2020, Elsevier.

Different 3D‐printing techniques including SLA, fused deposition modeling (FDM), selective laser sintering (SLS), and multijet fusion, have been used for fabricating piezoelectric generators and sensors.^[2]^ FDM technique has the advantage of high efficiency, easy operation, low cost, and less effect on environment and as the results, it is considered for printing of piezoelectric parts. One of the challenges of FDM printing for PVDF is less formation of *β*‐phase in compare with *α*‐crystals phase in molten state which directly affects the piezoelectric performance (**Figure** **7**a).^[^ ^138^ ^]^ The SLS 3D‐printing technique applies heat of laser on materials powder to sinter them layer by layer and make the final components (Figure 7b).^[^ ^139^ ^]^ Before sintering by SLS, filler and polymer should be fully mixed and prepared into powder, which offer development of new approaches for the dispersion of conductive fillers.^[^ ^140^ ^]^

In the SLA method, a photosensitive liquid exposed to the laser light and due to cross‐linking, the sold regions are formed. The time of light exposure, laser intensity, and pixel size of the screen affected the resolution quality of the structure and depend on the device properties. One of the methods to reach high resolution in SLA devices is using microscale digital projection printing (DPP), which leverages a digital micromirror‐array device to fabricate a dynamic digital mask (Figure 7c). In the DPP technique, due to the light exposure, piezoelectric nanoparticles are grafted to the polymer backbones in composite solution. This direct linkage leads to piezoelectric performance enhancement of fabricated 3D structure by applying mechanical stress to the piezoelectric crystal.^[^ ^141^ ^]^

The 3D printable extrusion ink technique forms multilayer stacking based on solvent evaporation during layer formation. An all‐3D printed piezoelectric energy harvester is fabricated from BaTiO_3_ NP/P(VDF‐TrFE) ink (Figure 7d). Conductive Ag flake/P(VDF‐TrFE) ink was printed on the top of the printed piezoelectric structure as the top electrode for the poling process.^[^ ^142^ ^]^ In most of the fabrication techniques, additional steps (painting conduction ink or paint, screen printing , sputtering , sticking conductive tape or fabrics ) are needed to add electrodes on both side of piezoelectric parts which make fabrication complicated and costly but 3D printing has feature of co‐fabricating of electrodes with piezoelectric part in a single manufacturing process.^[^ ^143^ ^]^ Nowadays numerous 3D‐printing techniques are easily accessible, but the considerable challenge for many printing processes is limitation in choosing printable material.^[^ ^132^ ^]^ Advantages of 3D printing in piezoelectric materials as sensors are customization, cost‐effectiveness, quick prototyping, and high accuracy in dimensional fabrication. However, the 3D‐printing technology has many challenges toward printing piezoelectric sensors including providing in situ poling, limitation on printable materials, providing high resolution for surface finish, and embedding electrodes which need further exploration. Also, the strength of piezoelectric sensors produced by 3D printing may be lower compared to those produced using traditional manufacturing techniques.

### 5.2 Electrospinning

Many studies confirmed that electrospinning is an accomplished technique to fabricate 1D nanofiber for sensors application.^[^ ^144^ ^]^ Electrospinning is a versatile, simple, fast, and efficient method to fabricate submicron or nanoscale fibers from polymer solutions.

**Figure 8:**
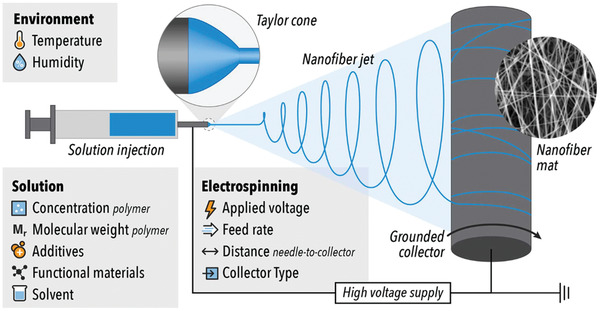
Schematic illustration of a typical electrospinning setup with a list of effective parameters on nanofiber properties. Reproduced with permission.^[^ ^146^ ^]^ Copyright 2022, Elsevier.

Nanofibers prepared by electrospinning have uniform morphology and large specific surface area.^[^ ^57^ ^]^ The key reason for improved piezoelectric performance of nanofibers is assigned to the physical and electrostatic forces applied to the polymer droplet on tip of needle during electrospinning. Taylor cone (**Figure** **8**) is a cone shape structure which is formed as the result of applying high amount of positive charge onto a small droplet of polymer solution and the creation of potential difference between needle tip and negative charge of collector during electrospinning. This stretching force between positive (needle tip) and negative charge (collector) in addition to the applied voltage leads to fiber polarization and *β*‐phase formation in fibers’ structure.^[^ ^9^ ^]^ Piezoelectric martials in the form of fiber or any other flexible format can be integrated to the textiles and detect a variety of human physiological signals and any environmental vibration to harvest their energy.^[^ ^145^ ^]^ The electrospinning approach with capability of fabricating breathable and flexible structures, opens a new direction for the advancement of portable and wearable devices for motion detection and healthcare monitoring.

**Figure 9:**
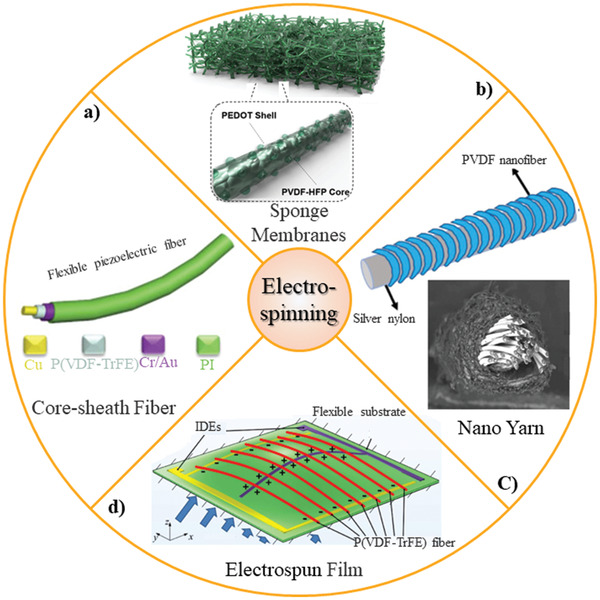
Different structure from electrospinning technique: a) P(VDF‐TrFE)‐based core–sheath structure smart fiber. Reproduced with permission.^[^ ^147^ ^]^ Copyright 2020, Elsevier, b) electrospun 3D nanostructured PVDF‐HFP/PEDOT nanofiber mats. Reproduced with permission.^[^ ^148^ ^]^ Copyright 2020, Nature, c) PVDF nanofiber wrapped around silver‐coated Nylon. Reproduced with permission.^[^ ^149^ ^]^ Copyright 2021, Wiley, d) schematic of the airflow sensor with showing piezoelectric fiber's electromechanical principle. Reproduced with permission.^[^ ^150^ ^]^ Copyright 2022, Elsevier.

A micron‐size stitchable piezoelectric fiber with core–sheath structure is fabricated by directly electrospinning the P(VDF‐TrFE) onto the flexible conductive wire (**Figure** **9**a). These fibers presented a high sensitivity of 60.82 mV N^−1^ and excellent durability under positive pressure over 15 000 cycles. Fibers were integrated into textiles to be worn and monitoring human motion.^[^ ^147^ ^]^ Electrospun pressure sensors have low performance in changing resistance with ambient pressure due to low porosity of fibers. Recently, novel hybrid 3D structure was fabricated, and the resulting structures featured a combination of high electronic conductivity and mechanical flexibility and durability. The 3D electrospinning and vapor deposition method were used to fabricate sponge‐like 3D membranes of (PVDF‐HFP)/poly(3,4‐ethylenedioxythiophene) (PEDOT) (Figure 9b). This sponge‐like web by increasing pore volume enhanced mechanoelectrical properties and exhibited a pressure sensitivity 13.5 kPa^−1^, which is higher than conventional electrospun mats (5.1 kPa^−1^).^[^ ^148^ ^]^ Nanofiber yarns demonstrated superb stress sensor performance due to taking advantage of both nanofibers and yarn's structure. PVDF nanofibers are wrapped around silver‐coated nylon yarns and formed a resilient and tough piezoelectric yarn (Figure 9c). The yarn with 600 twists per meter shows excellent piezoelectric properties with maximal *β*‐phase ratio, crystallinity, and piezoelectric voltage constant of 0.402, 49.57%, and 0.4323 mV m N^−1^, respectively.^[^ ^149^ ^]^ Airflow sensors are crucial components in many engineering systems including aerospace engineering, environmental monitoring, sustainable energy utilization, and weather forecasting. A piezoelectric sensing film was fabricated for calibration and detection of airflow velocity with lower detection limit of 0.3 m s^−1^ and the sensitivity of 15 mV m^−1^ s^−1^ (Figure 9).^[^ ^150^ ^]^ Various electrospinning methods may induce different piezoelectricity values of piezoelectric nanofibers, which is not compressively studied and needs more research effort.

Overall, using electrospinning technique to fabricate piezoelectric sensors has advantage of fabricating piezoelectric fibers with a high surface area, which enhances the sensitivity of the sensor. It also allows to produce piezoelectric fibers with different diameters and lengths, enabling customization of the sensor to meet specific requirements. This method is a cost‐effective option for producing piezoelectric sensors with high accuracy and precision. However, this method in addition to the complex setup and specialized equipment it needs, is a slow and time‐consuming process, which may limit its scalability for large‐scale production of piezoelectric sensors. The choice of materials for electrospinning is currently limited, indeed the mechanical properties of electrospun piezoelectric fibers may be weaker compared to bulk materials, which may impact the durability and lifespan of the sensor.

### 5.3 Melt Spinning

**Figure 10:**
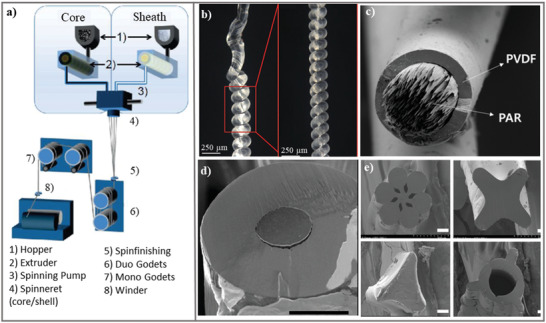
a) Melt spinning process for developing a core/sheath fiber. Adapted under the terms of the CC‐BY Creative Commons Attribution license.^[^ ^159^ ^]^ Copyright 2013, The authors, published by MDPI, b) coil formation process by inducing twist to the PVDF/rGO/BT melt spun fiber. Reproduced with permission.^[^ ^155^ ^]^ Copyright 2021, Wiley, c) SEM image of the PVDF/PAR core–shell fiber. Adapted under the terms of the CC‐BY Creative Commons Attribution license.^[^ ^156^ ^]^ Copyright 2020, The authors, published by MDPI, d) SEM image of the cross‐section of the core (carbon black/polyethylene) and sheath (PVDF) of melt spun fiber. Reproduced with permission.^[^ ^157^ ^]^ Copyright 2018, Nature, e) SEM image of melt spun PVDF fibers correlated with biomimetic cross‐sectional morphologies (daffodil, radish flower, papyrus stem, stalk grain stem). Reproduced with permission.^[^ ^158^ ^]^ Copyright 2022, Elsevier.

Melt spinning is an extrusion method for fabricating piezoelectric filaments in low cost and mass production. In the melt spinning process, the melted polymer is coming out of a die, be cooled by air, and then is collected on a drum (**Figure** **10**a). Filament crystallinity is affected by the tension applied on them between die and first take‐up roll which can be controlled by collector speed. Melt spinning of piezoelectric polymers causes a chain orientation and produces mainly nonpolar *α*‐phase over the filaments. Incorporation of fillers or posttreatment technique such as thermal drawing and electrical polarization can be used to increase the electroactive phase fraction (*β*‐phase formation) in piezoelectric filaments.^[^ ^151^ ^]^

Several characterization techniques are employed to measure the crystallinity of melt spun fibers including X‐ray diffraction, wide‐angle and small‐angle X‐ray scattering, and IR spectroscopy. The disadvantage of these studies is crystallinity evaluation after fiber formation, therefore needs to have a technique for in situ assessment along the process. Recently, research was conducted studying the evolution of the crystalline phases (*α*‐ and *β*‐phase) directly on a melt‐spinning pilot using Raman spectroscopy to understand which stage of the process leads to the transformation of the phases.^[^ ^152^ ^]^ The more control is over fiber diameter in melt spinning in compare with electrospinning, however melt spinning needs additional set up for stretching and poling as the posttreatment process.^[^ ^64^ ^]^ A novel approach was developed for fabrication of melt spun PVDF fibers with improved mechanical (i.e., durability, flexibility, and comfort) and piezoelectric properties (i.e., sensitivity and power output). In this method, a triaxial braided structure from PVDF filament and conductive silver‐coated nylon yarns were used to provide an efficient and novel way to overcome the stability issues due to the poor fatigue resistance of the metallic electrodes.^[^ ^153^ ^]^

The melt spun piezofiber with 98% of the electroactive *β*‐phase in a hybrid circular knitted structure of PVDF/BT demonstrated excellent performance in generating open circuit voltage of 4 V and a power density 87 µW cm^−3^ during cyclic compression.^[^ ^154^ ^]^ The melt spun fiber of PVDF/rGO/BT formed in the coil structure as a highly stretchable piezoelectric sensor and generator for motion detectors, and also for self‐powered biomedical applications (Figure 10b).^[^ ^155^ ^]^ PVDF and polyarylate (PAR) sheath–core fiber were fabricated by melt conjugate spinning which is faster and cheaper in compare with the sea‐island conjugate procedure. ZnO was coated on PVDF/PAR fibers through a surface growing method under various growth conditions to enhance the piezoelectricity of the fiber (Figure 10c).^[^ ^156^ ^]^ A core–sheath structure, where one electrode is hidden within the core of the fiber, was fabricated by melt spinning to exploit traditional manufacturing routes. In a practical example, this fiber was fabricated in a woven structure of 2.5 cm × 20 cm piece and used as the shoulder strap of a laptop case, which produced a continuous output of 4 µW (Figure 10d).^[^ ^157^ ^]^ The crystallinity and *β*‐phase formation in PVDF fibers is affected by cross‐sectional morphology due to differences in the exterior surface area and contact volume. PVDF fibers were fabricated by melt‐spinning with different cross‐sections inspired by plants (Figure 10e). Among different cross‐sections, the daffodil one generated highest voltage of 36.05 V due to its highest exterior surface area and contact volume that maximize the active area.^[^ ^158^ ^]^

Regarding the importance of melt spun piezoelectric fibers in energy harvesting and sensor application, it is required to utilize a standard for the measurement of electrical response and appropriate method for in situ characterization of the individual fibers.

Melt spinning is a high‐throughput manufacturing technique that can produce large quantities of piezoelectric fibers quickly and in a cost effective way which can be used with a wide range of materials. The fabricated fibers by this method have good mechanical properties, however fibers need posttreatment procedures such as poling and cold drawing to provide high piezoelectric performance.

## 6 Perspective and Future Outlook

Today's wearable technology has intensely changed our daily lives and developed an urgent demand for new and smart sensing technologies. Various vibration sources, including the vehicle and railway vibration, bridges and civil structures excited by earthquakes, wind, waves and other dynamic loads, human physiological movement, sound and ocean waves, and industrial machines considered as a power source for energy harvesting. Every industrial machine possesses vibrations and among them heavy machine at mining industry exposed workers to high level of vibration which has a huge threat to miner's health. This vibration is sourced from mining equipment, vehicles, and operational processes. The harvesting energy from vibration equipped with piezoelectric materials has been investigated for decades to supply sustainable power to wireless sensors. There are several high‐risk conditions for the workers in underground coal mines during their work, which may cause loss of life or serious injury that has a direct and indirect cost for employees and employers. Miners are regularly exposed to vibration levels which based on ISO 2631‐1 standard is over than safety limits.

Large‐scale mechanization considerably adds to the severity and complexity of the WBV problem because of a) increase in the percentage of exposed miners and b) longer durations of exposure. To examine miners’ individual health in real‐time and take necessary actions when the working environment is nonpreferable for the miner, the smart PPE is an urgent requirement. Providing PPE capable of detecting harmful vibration and issue alerts can minimize vibration adverse effect. Smart, digital, and appropriate PPE can help the mining industry in near future and allow for adequate occupational health and safety (OH&S) measures to be put in place. PPE is a part of wearable technology that with unique properties, e.g., light‐weight, soft nature, wearable convenience, and air permeability, can be an ideal candidate for future smart PPE. The challenges of safety, occupational health, and communication at mining sites have been successfully improved by wearable technologies. By using wearable sensors, the data of mine workers' health parameters (heartbeat, blood pressure, respiration rate, body temperature) and the coal mining environment vibration level can be transmitted to online services by means of IoT devices for active monitoring.

The battery life of wearable electronics is a key technology that limits their development. Li‐ion batteries have higher risk of fire or explosions due to potential battery failures within underground mining environments. The proposal of self‐powered wearable electronics provides a promising solution to the problem of long‐term stable working of wearable electronics. Self‐powering sensors are currently seen as critically important units for wearable and nonwearable textile–electronic systems. The piezoelectric acceleration sensors that use vibration energy to realize self‐powered and self‐sensing can be used to monitor the acceleration of vibrations that affect workers. The piezoelectric materials including piezoceramics and piezopolymers have been used to develop energy harvesting materials. The piezopolymers (i.e., PVDF) have distinct features of chemical stability and flexibility, which enable them to fabricate fiber‐based sensors for wearable technology applications. The piezoelectric properties of the PVDF polymer rely on the aggregate of *β*‐phase formation. During fabrication of piezoelectric sensors there are different methods to enhance their *β*‐phase including electrical poling, mechanical stretching, filler addition, annealing and cooling, and pressing. As PPE sensors, piezoelectric fiber‐based generators are in high demand due to their light weight and easy integration into textile products without additional load on miner workers.

Electrospinning and melt spinning are two common techniques for fabricating piezoelectric fiber. The electrospinning method delivers in situ poling on fabricated nanofibers which make them to be ready to use for final application without need to any posttreatment process. Their disadvantage is needing metallic/metal electrodes, which may affect their application in providing flexibility and long lifetime. The uniform fibers dimeter with low‐porosity is obtained by melt spinning process. However, the melt‐spun fibers require posttreatment including cold drawing and poling to align their molecular dipoles.

Depending on the final application of sensors, 3D printing is a trending technique in the area of printing technology for creating novel wearable platforms. 3D printing has revolutionized almost every manufacturing sector with complicated geometries that can be created in a layer‐by‐layer manner based on the available digital model. However, choosing appropriate printable material is a matter of concern in 3D‐printing technique to achieve a flexible, durable, and efficient wearable device.

Although a numerous research works has been carried out in relation to wearable piezoelectric generators and sensors, still several important problems remain and need to be tackled in the future:

1. First, piezoelectric generators are still in the experimental phase, and their durability in daily wear for the long‐time effect is not studied yet. Their function to the elements such as sweating, daily wash with detergent, or other problems associated with real‐world usage need to be explored.
2. New functional materials and different synthetic routes need to be developed to provide high energy conversion efficiency, well‐characterized polarizability, surface properties, and functionality to boost electrostatic interactions between piezopolymer chains and fillers.
3. The result of piezoelectric sensors (sensitivity) and generators (current, voltage, energy conversion efficiency, …) from different research groups are not comparable due to variety of testing methods and reporting data. It requires establishing a standard for testing and reporting performance of devices to speed up research progress in this field.
4. The intrinsic application of piezoelectric sensors is real‐time monitoring for a long period of time without the change in their sensitivity and accuracy or any deformation. The research challenge is developing materials and designing packaging techniques for the sensors to prevent deformation and enhance their durability and stability. Choosing conductive filler such as Maxene with superior mechanical properties as the filler, using silver‐coated nylon fiber as electrodes, and coating final sensor device with PDMS are some recent efforts in this field.
5. For future practical application of self‐powered piezoelectric sensors, they can be equipped with ML and remote control to be used in AI applications. Adding multiple functionalities to these sensors promotes their application for entire wireless sensor nodes.
6. Current textile‐based piezoelectric generators are mostly hand‐woven and in small size with lower output. The bigger size of generator which can be woven or knitted with textile machine needs to be studied in all aspects of performance, durability, and stability in the future.
7. With the growth and development of IoT, sensors networks face new challenges. The self‐powering sensors for real‐time monitoring are not easily accessible and need to be established and fabricated.

## 7 Conclusions

In this article, we have provided recent advances that have taken place in wearable energy harvesting for workplace OH&S—framed initially in the context of the mining and construction application domains. Novel piezoelectric energy harvesting materials have shown great potential for achieving viable application ready technology. We initially examined workers’ PPE (mandatory safety equipment) as platforms for the deployment of wearable energy harvesting systems and explored the reported state‐of‐the‐art capabilities in the literature. This is subsequently extended to opportunities of this material for structural and environmental monitoring. Latest advances of piezoelectric materials in ceramics, SCs, and polymers are later explored in addition to the fabrication of these materials via the application of 3D printing, electrospinning, and melt spinning. Overall, we find that the area of energy harvesting using piezoelectric materials is at a stage of maturity for providing exciting possibilities to overcome battery limitations and demonstrate their capabilities as wearable energy harvesters in hazardous workplace environments.

## Conflict of Interest

The authors declare no conflict of interest.
